# Supplementary material for: Are performance trajectories associated with relative age in French top 100 youth table tennis players? – A longitudinal approach
Source: PLoS One. 2020 Apr 21;15(4):e0231926. doi: 10.1371/journal.pone.0231926 (PMC7173848; doi:10.1371/journal.pone.0231926)
Supplement: S1 Table — (PDF) [file pone.0231926.s001.pdf]

S1 Table. Fit indices of Latent Class Growth Analysis (LCGA) models with 1–5 classes for subsamples.

		Females					*Males*				
		1 class	2 class	3 class	4 class	5 class	1 class	2 class	3 class	4 class	5 class
U14	Log likelihood	-5180.59	-4844.59	-4635.80	**-4530.56**	-4439.71	-5385.09	-5078.82	**-4955.87**	-4875.68	-4821.56
	AIC	10383.19	9719.19	9309.61	**9107.12**	8933.41	10792.18	10187.63	**9949.73**	9797.36	9697.11
	BIC	10411.85	9758.27	9359.10	**9167.04**	9003.75	10820.83	10226.71	**9999.23**	9857.28	9767.45
	LRT	-	672.01	417.58	**208.28**	181.704	-	612.59	**245.90***	190.78	108.25
U15	Log likelihood	-5311.63	-5001.35	-4818.57	**-4697.23**	-4594.99	-5569.20	-5314.70	-5152.38	**-5046.89**	-5005.69
	AIC	10645.25	10032.69	9675.14	**9449.47**	9243.98	11160.40	10659.40	10342.77	**10139.78**	10065.39
	BIC	10673.91	10071.77	9724.64	**9500.38**	9314.32	11189.06	10698.48	10392.27	**10199.70**	10135.73
	LRT	-	620.58	365.55	**255.77**	205.01	-	509.02	324.63	**210.99**	82.39
U16	Log likelihood	-5475.11	-5114.31	-4930.75	**-4794.50**	-4721.32	-5528.47	-5211.33	**-5056.15**	-4974.23	-4909.00
	AIC	10972.23	10258.62	9899.50	**9635.01**	9496.64	11078.95	10452.66	**10150.30**	9994.45	9872.01
	BIC	11000.88	10297.70	9949.00	**9694.92**	9566.98	11107.60	10491.74	**10199.80**	10054.37	9942.35
	LRT	-	721.67	367.12	**272.49***	146.37	-	634.36	**310.36***	163.85	166.69*
U17	Log likelihood	-5618.83	-5237.60	**-4978.99**	-4861.46	-4787.55	-5535.51	-5199.79	**-5009.94**	-4909.64	-4846.77
	AIC	11259.65	10505.21	**9995.98**	9768.92	9629.09	11093.01	10429.57	**10057.88**	9865.28	9747.64
	BIC	11288.31	10544.29	**10045.48**	9828.84	9699.43	11121.67	10468.65	**10107.38**	9925.20	9817.88
	LRT	-	762.46	**517.23***	235.06	147.83	-	671.53*	**379.70***	200.60	125.74
U18	Log likelihood	-5651.15	-5268.21	**-4972.20**	-4834.96	-4741.31	-5502.48	-5231.31	-5086.27	**-4966.65**	-4886.55
	AIC	11324.29	10566.42	**9982.40**	9715.91	9536.61	11026.95	10492.63	10210.55	**9979.29**	9827.11
	BIC	11352.95	10605.50	**10031.90**	9775.83	9606.95	11055.68	10531.70	10260.04	**10039.21**	9897.45
	LRT	-	765.88	**592.02***	274.49	187.30	-	542.35	290.08	**239.25**	160.19

Bold entries reflect selected model. -: not available. *p < .05. AIC=Akaike Information Criterion; BIC = Bayesian Information Criterion; ABIC=Adjusted BIC; LRT=Lo. Mendell and Rubin Likelihood Ratio Test.
